# Revisiting the approaches of psychotherapy in Ayurveda with Research Domain Criteria (RDoC) framework: a review

**DOI:** 10.1192/j.eurpsy.2024.792

**Published:** 2024-08-27

**Authors:** W. Upadhyaya, A. Iyer

**Affiliations:** ^1^Centre Ahimsa, Verrières-le-Buisson, France; ^2^Independent and unaffiliated researcher, London, United Kingdom

## Abstract

**Introduction:**

Recently there have been increased acceptance of complementary and alternative medicine (including traditional medicines) not only among laypersons but also various medical specialities. Ayurveda is one such, that originated at least in 3000 BC in the Indian subcontinent. Ayurveda aims at not only treating diseases but also maintaining optimum health. Psychiatry branch of Ayurveda recommends the use of both medicines and psychotherapy. Past papers on Ayurvedic psychotherapy have limitations in terms of semantics, conveying relevance and practical implementation. To tide over such limitations, we review concepts of psychotherapy in the Ayurveda texts Charaka Samhita (CS), Sushruta Samhita (SS), Ashtanga Hridaya (AH) and their commentaries from the original Sanskrit texts, in light of RDoC framework. The approaches derived can be used not just for therapy but also as mental health promotion.

**Objectives:**

1. To delineate approaches to psychotherapy from Ayurveda classics and their commentaries, which are useful for both mental health promotion and therapy.
2. To view the components of Ayurvedic psychotherapy approaches in terms of RDoC constructs/subconstructs.

**Methods:**

Relevant chapters were scanned in the texts CS, SS, AH and their commentaries for descriptions of psychotherapy. Consequently, its components were compared with the definitions of constructs and subconstructs of RDoC to identify similarities.

**Results:**

Only CS and AH had descriptions on psychotherapy, among which, one out of the four described in CS and the only one in AH was suitable for our purpose. The components of these models with relevant counterparts (single or combined) are tabulated in Table 1.

**Table 1:** 

CS psychotherapy model	RDoC construct/ subconstruct
1) Spiritual awareness (Jnana)	Declarative memory (semantic)
2) Specialised knowledge (Vijnana)	Declarative memory (semantic)
3) Self-control & equanimity (Dhairya)	Cognitive control
4) Memory (Smriti)	Declarative memory (episodic)
5) Meditative focus (Samadhi)	Attention, working memory
AH psychotherapy model	RDoC construct/ subconstruct
1) Intellect (Dhi)	Declarative memory.
2) Self-control (Dhairya)	Cognitive control
3) Knowledge of self and surrounding (Atmadi jnana)	Perception and understanding of self

**Conclusions:**

Thus, CS and AH provide a 5-dimensional and a 3-dimensional approach to psychotherapy respectively (with its components having correlates with few RDoC constructs or subconstructs) which can be explored clinically and evaluated, for therapy and mental health promotion purposes.

**Disclosure of Interest:**

None Declared

